# Extended Opioid Exposure Modulates the Molecular Metabolism of Clear Cell Renal Cell Carcinoma

**DOI:** 10.3390/life13051196

**Published:** 2023-05-17

**Authors:** Mamatha Garige, Sarah Poncet, Alexis Norris, Chao-Kai Chou, Wells W. Wu, Rong-Fong Shen, Jacob W. Greenberg, Louis Spencer Krane, Carole Sourbier

**Affiliations:** 1 Division of Biotechnology Review and Research 1, Office of Biotechnology Products, Office of Pharmaceutical Quality, Center for Drug Evaluation and Research, U.S. Food and Drug Administration, Silver Spring, MD 20993, USA; 2 Division of Animal Bioengineering and Cellular Therapies, Office of New Animal Drug Evaluation, Center for Veterinary Medicine, U.S. Food and Drug Administration, Rockville, MD 20852, USA; 3 Facility for Biotechnology Resources, Center for Biologicals Evaluation and Research, U.S. Food and Drug Administration, Silver Spring, MD 20993, USA; 4 Department of Urology, School of Medicine, Tulane University, New Orleans, LA 70112, USA

**Keywords:** RCC, opioids, metabolism, OXPHOS, TCA cycle, glycolysis

## Abstract

Opioids are commonly prescribed for extended periods of time to patients with advanced clear cell renal cell carcinoma to assist with pain management. Because extended opioid exposure has been shown to affect the vasculature and to be immunosuppressive, we investigated how it may affect the metabolism and physiology of clear cell renal cell carcinoma. RNA sequencing of a limited number of archived patients’ specimens with extended opioid exposure or non-opioid exposure was performed. Immune infiltration and changes in the microenvironment were evaluated using CIBERSORT. A significant decrease in M1 macrophages and T cells CD4 memory resting immune subsets was observed in opioid-exposed tumors, whereas the changes observed in other immune cells were not statistically significant. Further RNA sequencing data analysis showed that differential expression of KEGG signaling pathways was significant between non-opioid-exposed specimens and opioid-exposed specimens, with a shift from a gene signature consistent with aerobic glycolysis to a gene signature consistent with the TCA cycle, nicotinate metabolism, and the cAMP signaling pathway. Together, these data suggest that extended opioid exposure changes the cellular metabolism and immune homeostasis of ccRCC, which might impact the response to therapy of these patients, especially if the therapy is targeting the microenvironment or metabolism of ccRCC tumors.

## 1. Introduction

Clear cell renal cell carcinomas (ccRCC) are the most common types of kidney tumors, accounting for around 80% of all kidney cancers [1]. They are characterized, in most cases, by the mutation or loss of function of the von Hippel Lindau (VHL) tumor suppressor gene [2]. The VHL protein is part of a complex that regulates oxygen sensing within the cells and that drives the cell’s response to hypoxia by hydroxylating the transcription factor hypoxia inducible factor (HIF) when oxygen levels are low. Lack of the VHL leads to an aberrant stabilization and activation of HIF under normoxia and the activation of its downstream targets, such as the vascular endothelial growth factor (VEFG) or the glucose transporter 1 (Glut1) to promote angiogenesis and aerobic glycolysis, respectively [2]. Consequently, ccRCCs are highly immunogenic and vascularized and are characterized by an aberrant shift toward aerobic glycolysis [3]. The most common treatments for metastatic ccRCC disease involve therapies that promote immune infiltration of the tumor, target angiogenesis, or combine these mechanisms. Despite multiple recent approvals for novel medications for ccRCC, the survival rate for metastatic ccRCC remains poor [4,5]. Indeed, patients with advanced stages of ccRCC have a 5-year survival rate below 20%, underscoring the need to understand the efficacy and safety of current therapies. 

Opioid analgesics have been used to manage pain for a very long time, and numerous opioid analgesics have been used throughout the years [6]. They bind to the opioid receptors with differential binding affinity. The main three opioid receptors are µ (mu), δ (delta), and κ (kappa). Endogenous opioids such as enkephalins and β-endorphin are agonists of both the µ and the δ receptors, while exogenous opioids such as methadone and fentanyl are agonists of only the µ receptor. Exogenous opioids such as morphine, codeine, and meperidine activate the µ receptor as well as the κ receptor (morphine) and the δ receptor (codeine and meperidine). Naloxone and naltrexone are antagonists of both the µ and the κ receptors, with weaker binding to the δ receptor. Opioids are commonly prescribed to cancer patients, including kidney cancer patients, for extended periods of time to assist with pain management associated with metastatic ccRCC, particularly if the metastases cannot be resected and remain in place. It has been shown in other settings that long or chronic administration of opioids modulates the immune system, affecting both innate and induced immunity [7,8,9,10]. It could also affect tumor vascularization [11,12]. The molecular mechanisms underlying these effects have been broadly studied (as referenced above) and are thought to be mediated, at least in part, by opioid receptors located on immune cells, endothelial cells, and tumor cells. The μ-opioid receptor (MOR), in particular, has been shown to promote tumor growth via the activation of the mitogen-activated protein kinase (MAPK) and the extracellular signal-regulated kinases (Erk) pathway [13]. Some studies on ccRCC have looked at whether opioids could affect the aggressiveness of the tumors [14]. For example, Ma and collaborators have linked morphine-induced proliferation of ccRCC cell lines to the survivin signaling pathway. In addition, Scarpa and collaborators have recently identified several signaling networks proposed to be involved in the association between changes in opioid signaling and patient outcomes [15]. They showed that the pathway linked to fatty acid metabolism and the Th2 immune network, known to be involved in ccRCC growth, may be key players in mediating the effects of opioids [15]. Interestingly, the same study showed that the transcriptional expression of the genes coding for the opioids receptors µ (mu), δ (delta), and λ (lambda) were low in ccRCC tumors, suggesting that opioids might exert their effects via mechanisms independent of these opioid receptors in ccRCC. This is consistent with another study by Bisignani and collaborators that suggested that, in ccRCC cell lines, the opioid growth factor (OGF) receptor may mediate some of the opioid’s effects [16]; although in this case, addition of OGF inhibited the proliferation of the treated ccRCC cell lines in tissue culture. 

In this study, we hypothesized that prolonged opioid exposure might affect the metabolic profile of ccRCC. Changes in the metabolism of these tumors could have a significant effect on the tumors’ behavior. It could potentially modulate ccRCC aggressiveness or affect the efficacy of therapies targeting the immune system or angiogenesis of these tumors. To start addressing this question, we utilized archived formalin-fixed specimens of ccRCC that were either exposed or not exposed to opioids for more than 6 weeks and performed RNA sequencing to analyze whether the metabolic signaling pathways and immune infiltrate profiles were changed by the extended exposure to opioids. 

## 2. Materials and Methods

### 2.1. Criteria to Select Patients’ Specimens

The specimens analyzed were formalin-fixed paraffin-embedded (FFPE) samples from patients with untreated (naïve) clear cell kidney tumors. Tissue specimens were obtained in blocks from the primary tumor (nephrectomy or partial nephrectomy) at the time of the procedure. Archived samples were used for this retrospective study to allow for the processing and analysis of all the samples at the same time. 

The “extended opioid exposure” group consisted of specimens from patients who had been taking opioids for chronic pain for a duration of at least 6 weeks. Importantly, these patients were not receiving opioid therapies for cancer-related pain, but instead were on these medications due to chronic pain unrelated to their renal malignancies. 

All patients who were selected for this study had clinically localized ccRCC, and as standard of care did not include neoadjuvant treatment in this patient cohort, none had received therapy prior to surgical extirpation. Specimens of similar grade, size, sex, and histology (ccRCC) were selected and anonymized. 

### 2.2. RNA Sequencing

Total RNA was extracted using the RNeasy FFPE Kit from Qiagen (Germantown, MD, USA) according to the manufacturer’s instructions and assessed for quality using the Agilent Bioanalyzer Total RNA Nano Chip (Agilent, Santa Clara, CA, USA). Samples with DV200 > 20 were selected to proceed for the RNA sequencing. An amount of 100 ng of total RNA was used to generate the RNA sequencing paired-end library through the Illumina RNA Prep with Enrichment Kit. RNA was reverse transcribed into cDNA; this was followed by fragmentation, probe hybridization, and individual indexing. The indexed cDNAs were enriched by exome probes and followed by 14 PCR cycle amplifications. After obtaining the sequencing libraries, 0.6 nM of the pooled library was subjected to sequencing by an Illumina NovaSeq instrument with SP capacity (Illumina, San Diego, CA, USA). Following the instruction provided by the manufacturer, paired-end 2 × 75 cycles sequencing was performed. Base calling was performed using the bcl2fastq program, producing adaptor-trimmed fastq files used for subsequent data analysis. 

Sequencing reads and gene analyses were performed as previously described [17]. Cell-type profiling was performed using CIBERSORT (http://cibersort.stanford.edu/; accessed 6 September 2020 [18,19]). Using the gene expression data (logCPM) and the LM22 default CIBERSORT dataset with 1000 permutations and quantile normalization disabled, CIBERSORT generated the fractional representations of each cell type present [18,19]. Statistical analyses were performed using R version 3.6.3.

Datasets are publicly available at GEO with accession number GSE214563.

## 3. Results

### 3.1. Selection and Preparation of Archived ccRCC Specimens for RNA Sequencing

Twenty anonymized specimens from twenty independent ccRCC patients were selected to be analyzed as part of this study. Ten specimens were from ccRCC patients with extended opioid exposure, and ten specimens were from ccRCC patients who did not receive extended opioid therapy. None of the patients had received any previous therapies. Extraction of RNA from the 20 archived formalin-fixed paraffin-embedded (FFPE) specimens was performed as described by the manufacturer’s instructions for the RNeasy FFPE Kit (Qiagen, see also the Section 2). However, as RNA degrades over time, the minimal quality necessary to perform further analysis was not reached for nine of the specimens. Therefore, only five opioid-exposed specimens and six non-exposed specimens were used for RNA sequencing and subsequent analysis. The sequencing data was then subjected to differential gene expression and KEGG geneset enrichment analyses, followed by CIBERSORT cell-type analysis, as previously described [18,19].

### 3.2. Metabolic Effects of Extended Opioid Exposure on ccRCC Specimens by RNA Sequencing

**Figure 1:**
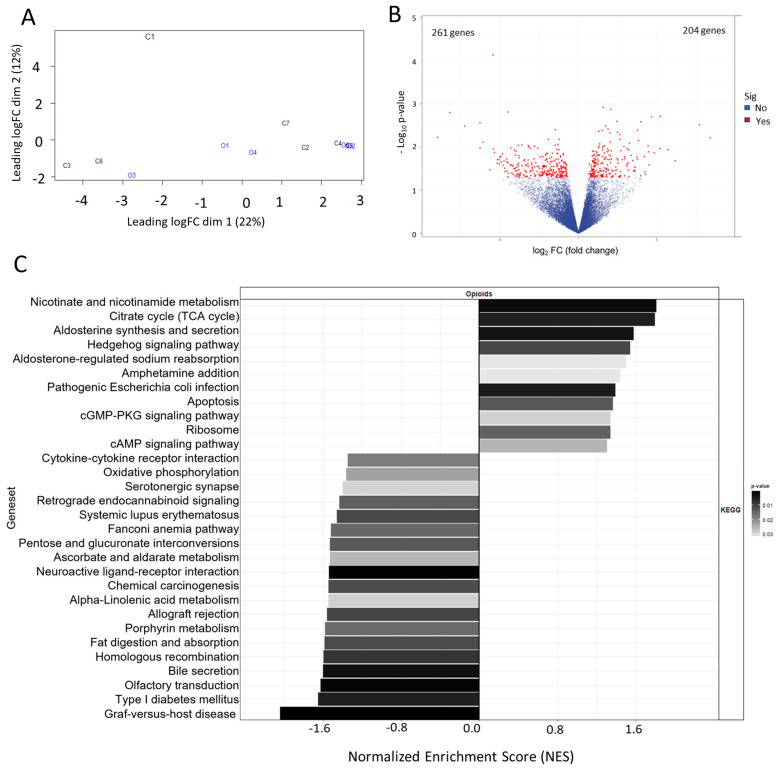
Metabolic effects of extended opioid exposure on ccRCC specimens by RNA sequencing. (**A**,**B**) MDS (multidimensional scaling) and volcano plots of the transcriptional profiles of opioid-exposed and non-exposed specimens following RNA sequencing. Significance was defined as FDR < 0.05 and *p* < 0.05. (**C**) KEGG geneset analysis shows signaling pathways that are differentially expressed in the opioid specimens compared with the naïve specimens, including TCA cycle, and nicotinate and nicotinamide metabolism; bars are shaded to indicate *p*-value from low [black] to high [white].

The effect of extended opioid exposure on the transcriptional profile of opioid-exposed and non-exposed specimens was assessed by RNA sequencing analysis. Transcriptional differences between the different groups (opioid-exposed vs. not opioid-exposed) were identified as illustrated by the multidimensional scaling (MDS) in Figure 1A (refer to Supplementary Table S1 for differential gene expression data for all genes). A total of 465 genes exhibited significant differential expression in opioid-exposed specimens compared with non-exposed specimens: 261 genes were down-regulated, and 204 genes were up-regulated, as shown in Figure 1B. The main 30 KEGG signaling pathways that were differentially activated between the two groups, based on the RNA sequencing data, are shown in Figure 1C and Supplementary Table S2. For example, the tricarboxylic acid cycle (TCA) cycle, the pathway of nicotinate and nicotinamide metabolism, the cAMP signaling pathway, and the pathway related to aldosterone-regulated sodium reabsorption presented transcriptional signatures consistent with an increased activation in tumors in patients exposed to opioids compared with non-exposed specimens. In addition, pathways related to lipid metabolism and Type I diabetes mellitus appeared to be down-regulated in opioid-exposed compared with non-exposed specimens (Figure 1C). These data demonstrate that tumors of ccRCC patients with extended opioid exposure present significant transcriptional differences when compared to non-exposed ccRCC specimens. These differences include the modulation of key metabolic pathways of these tumors. These data suggest that extended opioid exposure might play a role (directly or indirectly) in the modulation of these metabolic pathways.

**Figure 2:**
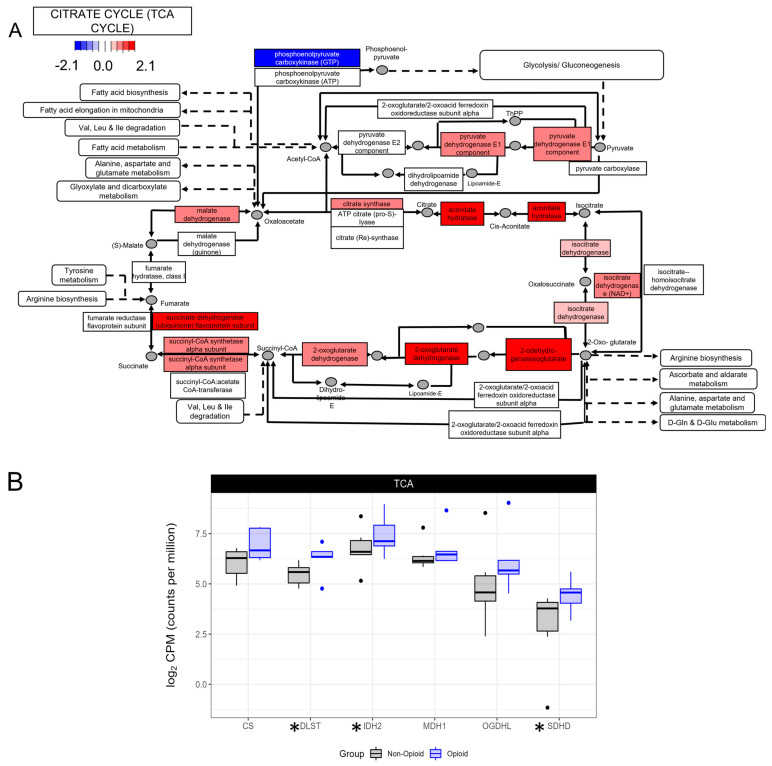
KEGG pathway analysis of the TCA cycle. (**A**) Enzymes in red have increased expression in the opioid specimens compared to the naïve. Expression of the enzymes in blue is decreased. Values are log2-transformed fold-change values between the opioid specimens compared with the naïve specimens; (**B**) boxplots representing the mRNA expression of selected members of the TCA cycle (DLST: dihydrolipoamide S-succinyltransferase; IDH2: isocitrate dehydrogenase; MDH1: malate dehydrogenase 1; OGDHL: oxoglutarate dehydrogenase like; SDHD: succinate dehydrogenase complex subunit D). * indicates that the gene is significantly regulated between the 2 groups (*p* < 0.05).

To further analyze the metabolic effects of extended opioid exposure on ccRCC specimens, we looked at the gene expression of some of the enzymes that are part the KEGG pathways which are shown to be significantly different between opioid-exposed and non-exposed specimens in Figure 1. Enzymes of the TCA cycle, such as aconitase hydratase, isocitrate dehydrogenase, the subunit alpha of succinyl-CoA synthetase, or malate dehydrogenase, were significantly up-regulated in opioid-exposed compared with non-exposed specimens, as shown in Figure 2A,B. This broad increase in mRNA expression was observed for almost all of the TCA cycle enzymes, with the exception of the fumarate hydratase enzyme for which expression was not significantly different between the opioid-exposed group and the non-opioid-exposed group. These data show that the opioid-exposed specimens included in this analysis have a transcriptional profile consistent with elevated oxidative phosphorylation.

**Figure 3:**
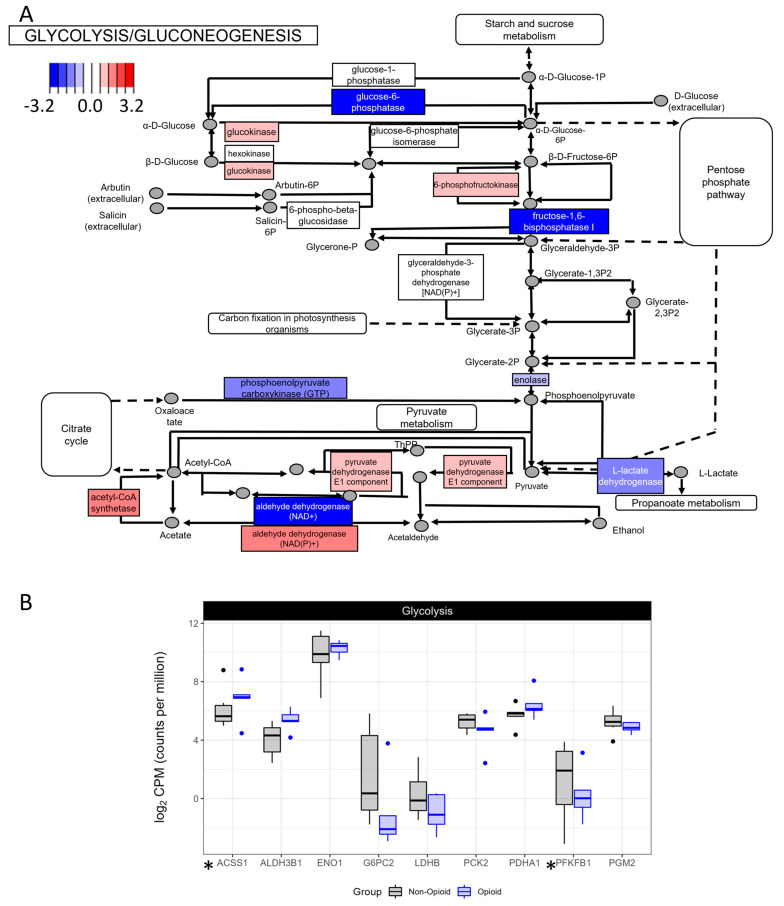
KEGG pathway analysis of glycolysis and gluconeogenesis. (**A**) Enzymes in red have increased expression in the opioid specimens compared to the naïve. Expression of the enzymes in blue is decreased. Values are log2-transformed fold-change values between the opioid specimens compared with the naïve specimens. (**B**) Boxplots representing the mRNA expression of selected members of glycolysis (ACSS1: acyl-CoA synthetase short chain family member 1; ALDH3B1: aldehyde dehydrogenase 3 family member B1; ENO1: enolase 1; G6PC2: glucose-6-phosphatase catalytic subunit 2; LDHB: lactate dehydrogenase B; PCK2: phosphoenolpyruvate carboxykinase 2; PDHA1: pyruvate dehydrogenase E1 alpha 1 subunit; PFKFB1: 6-phosphofructo-2-kinase/fructose-2,6-biphosphatase 1; PGM2: phosphoglucomutase 2). * indicates that the gene is significantly regulated between the 2 groups (*p* < 0.05).

An active oxidative phosphorylation is commonly in parallel with a decrease in glycolysis within a cell [20]. In concordance with the findings shown in Figure 2, the mRNA expression of several enzymes of the glycolysis/gluconeogenesis pathway were down-regulated, including the mRNA of fructose-1,6-biphosphatase 1, glucose-6-phosphatase, enolase, phosphoglucomutase 2, L-lactate dehydrogenase, and phospho-enol pyruvate carboxykinase 2 (Figure 3A,B). We also noted that the enzymes acetyl-CoA-synthetase, pyruvate dehydrogenase, and aldehyde dehydrogenase were all up-regulated (Figure 3A,B and Supplementary Table S1), which is consistent with an up-regulation of the TCA cycle. These data suggest that pyruvate metabolism and aerobic glycolysis, key features of ccRCC tumors, are down-regulated in ccRCC specimens following extended exposure to opioids in comparison to non-exposed tumors. 

**Figure 4:**
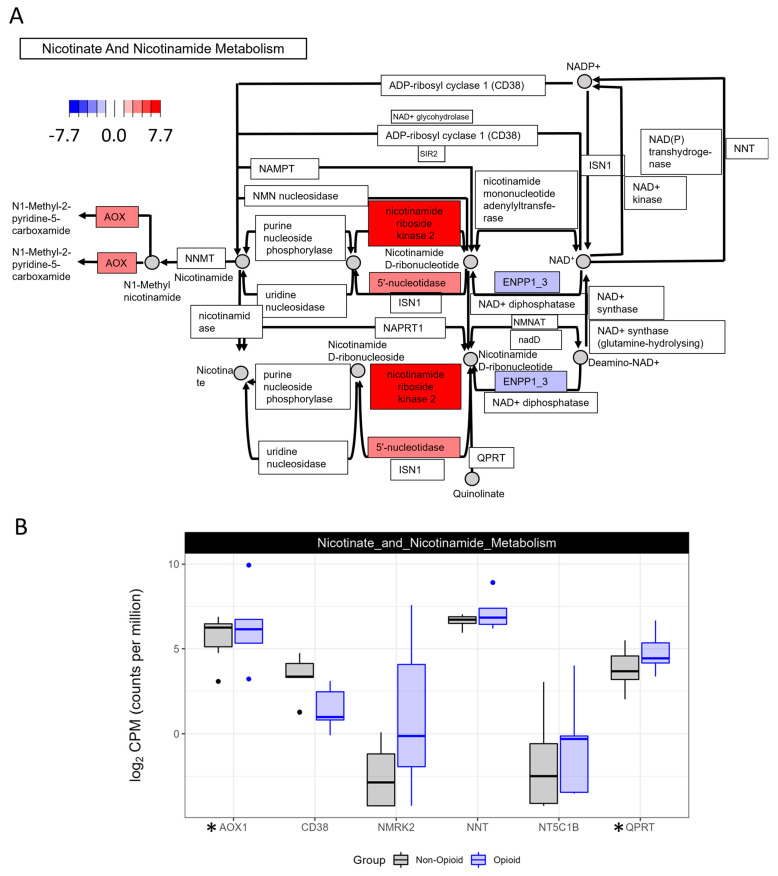
KEGG pathway analysis of nicotinate and nicotinamide metabolism. (**A**) Enzymes in red have increased expression in the opioid specimens compared to the naïve. Expression of the enzymes in blue is decreased. Values are log2-transformed fold-change values between the opioid specimens compared with the naïve specimens. (**B**) Boxplots representing the mRNA expression of selected members of nicotinate and nicotinamide metabolism (AOX1: aldehyde oxidase 1; CD38; NMRK2: nicotinamide riboside kinase 2; NNT: nicotinamide nucleotide transhydrogenase; NT5C1B: 5′-nucleotidase, cytosolic IB; QPRT: quinolinate phosphoribosyltransferase). * indicates that the gene is significantly regulated between the 2 groups (*p* < 0.05).

Consistent with the idea that opioid-exposed tumors switch their metabolism towards oxidative phosphorylation, the pathway of nicotinate and nicotinamide metabolism and the cAMP signaling pathway were both up-regulated in opioid-exposed compared with non-exposed specimens [21,22]. NAD+ and NADP+ are cofactors in multiple reactions within the mitochondria, and the ratios of their respective reduced forms regulate the activation of several metabolic enzymes [22]. The cAMP signaling pathway is important for maintaining mitochondrial function, in part, by activating PKA [21]. As shown in Figure 4 and Supplementary Table S1, the mRNA expressions of enzymes nicotinamide riboside kinase 2, aldehyde oxidase 1 (NT5C1B) and quinolinate phosphoribosyltransferase were up-regulated, whereas ADP-ribosyl cyclase 1 (CD38) was down-regulated in opioid-exposed ccRCC specimens.

**Figure 5:**
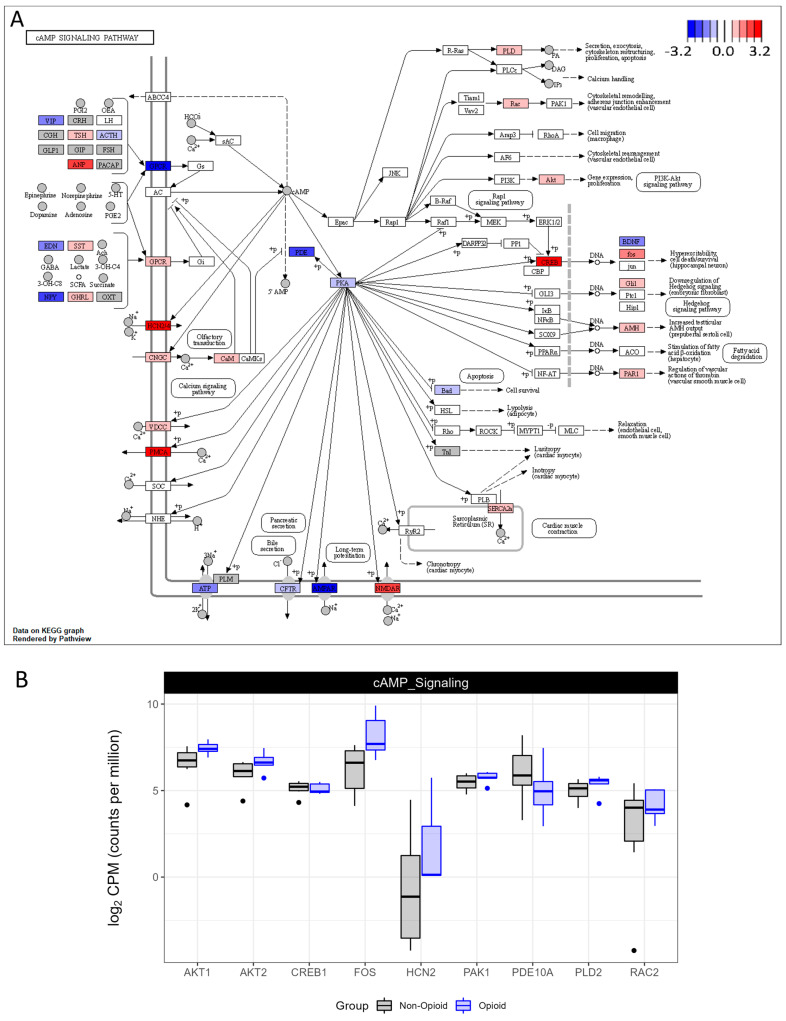
KEGG pathway analysis of cAMP signaling pathway. (**A**) Enzymes in red have increased expression in the opioid specimens compared to the naïve. Expression of the enzymes in blue is decreased. Values are log2-transformed fold-change values between the opioid specimens compared with the naïve specimens. (**B**) Boxplots representing the mRNA expression of selected members of cAMP signaling pathway (AKT1; AKT2; CREB1: cAMP-responsive element-binding protein 1; FOS; HCN2: hyperpolarization-activated cyclic nucleotide-gated potassium and sodium channel 2; PAK1: p21 (RAC1)-activated kinase 1; PDE10A: phosphodiesterase 10A; PLD2: phospholipase D2; RAC2).

Similarly, downstream effectors of the cAMP signaling pathway, such as cAMP-responsive element-binding protein (CREB), AKT serine/threonine kinase 1 (AKT1), AKT serine/threonine kinase 2 (AKT2), Fos proto-oncogene, AP-1 transcription factor subunit (FOS), HCN2 (hyperpolarization-activated cyclic nucleotide-gated potassium and sodium channel 2), phosphodiesterase10A (PDE10A), p21 (RAC1)-activated kinase 1 (PAK1), and phospholipase D2 (PLD2), were up-regulated in opioid-exposed compared with non-exposed specimens (Figure 5A,B and Supplementary Table S1). These data suggest that nicotinamide metabolism is increased in opioid-exposed tumors, which further supports the hypothesis that ccRCC tumors switch their metabolism towards oxidative phosphorylation following extended opioid exposure. In addition, since both the nicotinamide and the cAMP signaling pathways are known to promote the activation of sirtuins, key regulators of genome integrity [23,24], our data suggest that opioid-exposed ccRCC specimens might be subjected to epigenetic changes.

### 3.3. Immune Effects of Extended Opioid Exposure on ccRCC Specimens

**Figure 6:**
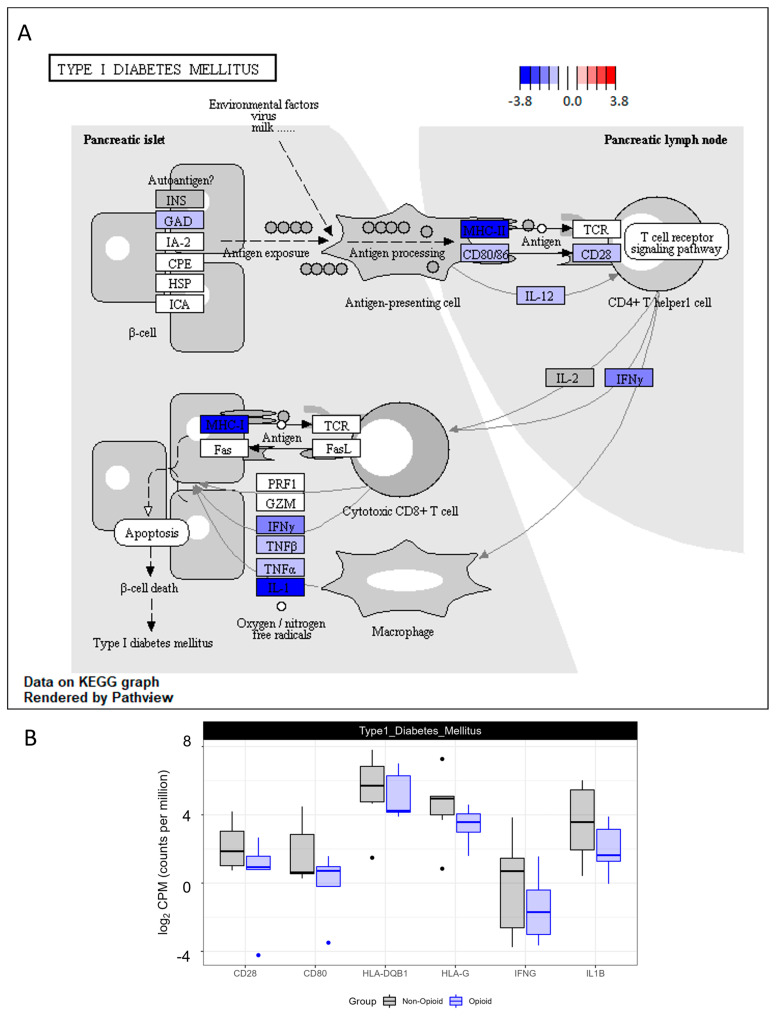
KEGG pathway analysis of Type I diabetes mellitus pathway. (**A**) Enzymes in blue have a decreased expression in the opioid specimens compared to the naïve. Values are log2-transformed fold-change values between the opioid specimens compared with the naïve specimens. (**B**) Boxplots representing the mRNA expression of selected members of type I diabetes mellitus pathway.

One of the signaling pathways that was down-regulated in ccRCC opioid-exposed tumors is the Type I diabetes mellitus pathway (Figure 6). Type I diabetes mellitus is a chronic life-threatening disease due to the degradation of the β-cells of the pancreas, the cells that make insulin. The destruction of the pancreatic β-cells is thought to be due to an unregulated immune response and leads to the incapability of the body to generate insulin, a peptide hormone involved in glucose uptake regulation, adipose triglyceride breakdown, and energy metabolism regulation [25,26]. The mRNA expression of genes affiliated with Type I diabetes, such as major histocompatibility complex class I and II, CD80 and CD28 molecules, interferon gamma (IFNγ), interleukin 1 (IL-1), and tumor necrosis factor alpha/beta (TNF), were all down-regulated in opioid-exposed ccRCC tumors compared to non-exposed ccRCC specimens. The modulation of the gene transcription of the members of this immunogenic signaling pathway suggests that extended exposure of ccRCC tumors to opioids may affect their immune composition and potentially their tumor microenvironment. 

**Figure 7:**
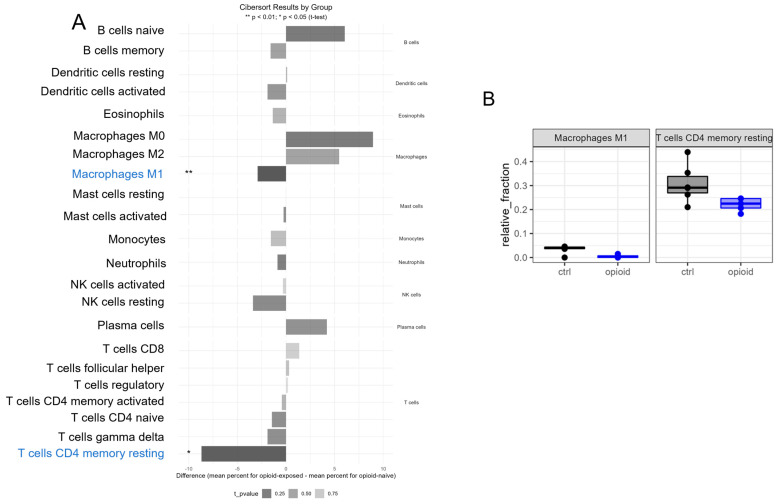
Immune effects of extended opioid exposure on ccRCC specimens by RNA sequencing. (**A**) CIBERSORT software was used to profile immune infiltrates using RNA sequencing data. (**A**) Bars toward the right indicate an increased expression in the opioid-exposed samples compared to naïve tumors. Bars toward the left indicate a decreased expression. (**B**) Statistically significant changes were identified as *p* < 0.05; bars are shaded to indicate *p*-values from 0 [black] to 1 [white].

Opioids have been shown to directly and indirectly modulate both innate and adaptive immunity [7,8,9,10]. Therefore, we sought to further investigate the potential immune effects of extended opioid exposure on ccRCC tumors. Using CIBERSORT software [18], we assessed whether the immune infiltration signature of the archived specimens was different between the opioid-exposed and the non-exposed ccRCC groups. The transcriptional profiles of immune infiltrates present in the ccRCC specimens were extracted from the RNA sequencing data of opioid-exposed and non-exposed specimens (Figure 7). The relative expression between groups of immune cells by cell type is shown in panel A of Figure 1. Opioid-exposed tumors presented a trend towards a more immunosuppressive immune infiltrate phenotype, with M1 macrophages and T cells CD4 memory resting presenting a statistically significant decreased expression (defined as *p* < 0.05) in opioid-exposed ccRCC specimens compared with non-exposed ccRCC specimens, as shown in Figure 7A,B. These data show that extended opioid exposure was immunosuppressive and affected the microenvironment of the tumor specimens that we assessed.

## 4. Discussion

In this study, we explored the effects of extended opioid exposure in ccRCC patients who received opioid therapy for over 6 weeks on the metabolism and immune landscapes of their ccRCC tumors using a limited number of formalin-fixed archived specimens. Based on our dataset, expended opioid exposure appears to modulate key metabolic pathways of the ccRCC specimens that were analyzed. These samples displayed a transcriptional profile consistent with a switch towards oxidative phosphorylation and an up-regulation of the nicotinate and nicotinamide metabolism and cAMP signaling pathways that could modulate genome integrity through the activation of sirtuins and lead to epigenetic changes. In addition, our data showed that ccRCC tumors exposed to opioids were immunosuppressed when compared to the non-exposed specimens. Taken together, our data show that opioids significantly affect the metabolism and genomic integrity of the ccRCC tumors tested. Further studies with additional samples will, however, be necessary to be able to generalize these findings to all individuals with ccRCC, especially for patients with advanced ccRCC. Having limited samples might indeed cause analysis bias, and additional specimens with defined parameters, such as grade or the length of archiving, would need to be analyzed to expand these results to a broad population. This study provides a strong foundation to further assess the effects of extended opioid use on the metabolism and physiology of ccRCC and provides evidence that a retrospective study using formalin-archived specimens could be technically doable. 

Despite known severe adverse events, such as respiratory depression or addiction, and because of a lack of effective alternatives for severe pain management, opioids are the most widely used type of analgesic for treating severe pain, in particular, during the clinical care of cancer patients, such as patients with ccRCC. Until efficient non-opioid analgesics are available to manage severe pain, it is important to assess any impact of the extended use of opioids on tumor physiology and microenvironment. Metabolic transformation of tumors, such as the Warburg effect, was introduced as one of the eight hallmarks of cancer in 2011 [27,28]. Immune and stromal cells present in the microenvironment play a key role in supporting this transformation [20,27,29]. Kidney tumors are characterized by several genetic mutations and have numerous phenotypes that have shown differences in metabolic reprograming depending on their subtypes [30,31]. Clear cell RCCs are characterized by a metabolic shift towards aerobic glycolysis with increasing grade [3]. Clear cell RCCs are also known to be highly immunogenic [32] and to have an increased vasculature [33]. Most renal malignancies are diagnosed incidentally, and with increasing rates of opioid use for the treatment of non-cancer pain or cases of opioid addiction, patients with long-term opioid exposure unrelated to their malignancy may have tumors with unique or untraditional metabolomic signatures, which may affect their response to therapy. Therefore, understanding how extended opioid exposure affects the immune profile and physiology of ccRCC would indicate to us whether further studies are needed to assess how opioids may affect the therapeutic efficacy of therapies targeting these pathways.

It is common for patients with metastatic ccRCC to receive, as a first-line therapy, a therapeutic agent targeting the vasculature. These agents include sunitinib, pazopanib, or bevacizumab, with sunitinib and pazopanib being the most used treatments for good- and intermediate-risk patients [1,33,34]. It is also worth noting that even though opioid exposure has been linked to changes in vasculature [11], we did not observe any effects on vasculature in our study (Supplementary Table S1). However, we found significant changes between the signaling pathways of tumors that were exposed to opioids and tumors that were not exposed (Figure 1, Supplementary Table S1). Key signaling pathways that were affected were related to metabolic processes, which suggests that extended opioid exposure might reshape the metabolic profile of ccRCC tumors towards less aerobic glycolysis and more oxidative phosphorylation. In addition, acetyl CoA synthetase, the nicotinamide pathway, and the cAMP signaling pathway were up-regulated in opioid-exposed specimens (Figure 3, Figure 4 and Figure 5), suggesting that chromatin remodeling might also occur in these tumors through activation of histone deacetylases, such as sirtuins [23,24]. The cAMP pathway has an ambiguous role in tumors, having been reported to have either tumor-suppressive or tumor-progressive roles [35]. In this study, opioid-exposed tumors presented an up-regulated cAMP signaling pathway and up-regulated downstream effectors, as seen in Figure 1 and Figure 5, that could affect tumor growth, migration, invasion, and metabolism. These data warrant further studies to better understand the role of cAMP signaling in patients with opioid treatment. Although we do not know from these data whether the different changes observed are promoting tumor growth or not, the therapeutic response may be altered following prolonged opioid treatment, and consideration of therapeutic regiments may need to be altered for these patients. 

Opioid exposure is known to affect immune homeostasis [7,8,9,10], and in concordance with numerous reports, we also observed a modulation of tumor immune infiltration in the tumors that were exposed to opioids for an extended period of time. The down-regulation of MHC class I and II in extended opioid-exposed ccRCC tumors suggests a decrease in the immunogenic signals of antigen-presenting cells to activate CD4+ T cells, which may lead to a decrease in the production of IL-2 and IFNγ and a reduced activation of cytotoxic CD8+ T cells/macrophages in opioid-exposed tumors (Figure 6). These data indicate that opioid-exposed ccRCC tumors might be more immunosuppressive than naïve ccRCC specimens. To further understand the effects of opioids on immune infiltration in ccRCC, we profiled the immune infiltrates present in opioid-exposed ccRCC specimens and non-exposed ccRCC specimens using CIBERSORT (Figure 7). The transcriptional profiles of both M1 macrophages and T cells CD4 memory resting were decreased in specimens that received extended opioid exposure compared with the non-exposed ccRCC specimens. Some reports have shown that opioids can induce broad changes in the tumor microenvironment [36,37]. Our data, however, show a more limited effect. This difference could be because the samples used for this analysis were formalin-archived, which might have affected the integrity of the tumors’ microenvironment. Another potential explanation could be that in ccRCC tumors, it has been shown that key opioid receptors such as the µ-opioid receptor (MOR) are not highly expressed, which could decrease the difference in amplitude of the results [14]. However, our data, similar to other reports, showed that extended opioid exposure was immunosuppressive and affected the microenvironment of the tumor specimens we assessed. In the tumor microenvironment, M1 macrophages recruit CD8+ T and NK cells by presenting antigens to the T-cell receptor and have been shown to suppress tumor cell growth [38,39,40]. According to Kovaleva and collaborators [41], in RCC, a low density of M1 (CD11+) macrophages and high density M2 macrophages were associated with poor survival. In addition, high levels of resting memory CD4+ T cells in ccRCC have been associated with improved outcomes because these cells can further differentiate into CD8+ T cells to suppress tumor growth [42]. It is important to note that our data are not linked to overall survival of ccRCC patients, and our study was not designed to answer this question. However, our data strongly suggest that extended opioid exposure induces an immunosuppressive environment. Further assessment of the potential effect of opioids on the tumor microenvironment of ccRCC would certainly help to understand the efficacy and safety of ccRCC therapies. Immunotherapies such as immune checkpoint inhibitors are now commonly used as first- or second-line approaches for ccRCC patients with advanced disease [4,32,43]. 

## 5. Conclusions

This study showed that, in a limited set of specimens, extended opioid exposure led to transcriptional changes consistent with the metabolic rewiring of localized ccRCC tumors towards oxidative phosphorylation and, potentially, epigenetic changes. Our data suggest that management of ccRCC patients with extended opioid therapy could significantly affect the molecular and cellular metabolism of these tumors as well as their microenvironments. Further studies will be necessary to generalize these findings to all patients with ccRCC. 

## Data Availability

All data generated or analyzed in this study are included in this published article and its Supplementary Materials. The accession numbers of the datasets used can be found in the article (Section 2).

## Supplementary Materials

The following supporting information can be downloaded at https://www.mdpi.com/article/10.3390/life13051196/s1, Table S1: Differential gene expression data file; Table S2: KEGG geneset enrichment data file.

## Abbreviations

CREB	cAMP-responsive element-binding protein
ccRCC	clear cell renal cell carcinoma
Erk	extracellular signal-regulated kinases
FFPE	formalin-fixed paraffin-embedded
Glut1	glucose transporter 1
HIF	hypoxia inducible factor
IL-1	interleukin 1
IFNγ	interferon gamma
MAPK	mitogen-activated protein kinase
MDS	multidimensional scaling
OGF	opioid growth factor
TCA	tricarboxylic acid cycle
TNF	tumor necrosis factor alpha/beta
VEFG	vascular endothelial growth factor
VHL	von Hippel Lindau
